# Northern Hemisphere vegetation change drives a Holocene thermal maximum

**DOI:** 10.1126/sciadv.abj6535

**Published:** 2022-04-15

**Authors:** Alexander J. Thompson, Jiang Zhu, Christopher J. Poulsen, Jessica E. Tierney, Christopher B. Skinner

**Affiliations:** 1 Department of Earth and Environmental Sciences, University of Michigan, Ann Arbor, MI 48109, USA.; 2 Climate and Global Dynamic Laboratory, National Center for Atmospheric Research, Boulder, CO 80305, USA.; 3 Department of Geosciences, The University of Arizona, Tucson, AZ 85721, USA.; 4 Department of Environmental, Earth and Atmospheric Sciences, University of Massachusetts Lowell, Lowell, MA 01854, USA.

## Abstract

The Holocene thermal maximum, a period of global warmth evident in early to mid-Holocene proxy reconstructions, is controversial. Most model simulations of the Holocene have not reproduced this warming, leading to a disagreement known as the Holocene Temperature Conundrum. Pollen records document the expansion of vegetation in the early and mid-Holocene African Sahara and Northern Hemisphere mid- and high latitudes, which has been overlooked in previous modeling studies. Here, we use time slice simulations of the Community Earth System Model to assess the impact of Northern Hemisphere vegetation change on Holocene annual mean temperatures. Our simulations indicate that expansion of Northern Hemisphere vegetation 9000 and 6000 years ago warms Earth’s surface by ~0.8° and 0.7°C, respectively, producing a better match with proxy-based reconstructions. Our results suggest that vegetation change is critical for modeling Holocene temperature evolution and highlight its role in driving a mid-Holocene temperature maximum.

## Teaser

Northern Hemisphere vegetation change is critical for the reconstruction of Holocene surface temperatures.

## INTRODUCTION

Paleoclimate reconstructions based on proxy records alone, including those of Marcott *et al.* (*1*), Shakun *et al.* (*2*), and, more recently, the Temperature12k database (hereafter referred to as T12K) (*3*, *4*), suggest a gradual cooling of ~0.5°C from the Holocene thermal maximum (HTM) in the early to mid-Holocene [~8 to 6 ka BP (thousand years before present)] to the preindustrial era (PI). In contrast, transient climate model experiments generally simulate global warming of ~0.5°C through the Holocene (*5*). The warming trend shown in transient climate model experiments (*5*–*7*) is a predictable response to declining ice cover, modestly increasing greenhouse gas (GHG) concentrations, and orbital-induced variations in insolation. The model-data mismatch, termed the “Holocene Temperature Conundrum,” potentially exposes uncertainty in our understanding of the ways in which the climate system responded to changes in forcings during the Holocene (*5*, *8*, *9*). While the HTM appears in global compilations of terrestrial and marine proxy records (*3*), as well as in regional compilations of Northern Hemisphere (NH) proxies interpreted as annual temperature (*10*, *11*), the conundrum also highlights the challenges of reconstructing global temperature trends from unevenly distributed proxy networks. Recent paleoclimate reconstructions based on data assimilation and tropical sea surface temperature (SST) records show no evidence of mid-Holocene warmth, raising the possibility that the HTM could potentially be an artifact of seasonal or spatial biasing (*12*, *13*).

While the existence of an HTM remains unresolved, if there was indeed a global HTM, then it suggests that additional relevant climate forcings may not have been accounted for in model simulations. Some previous studies have examined the role of dust (*14*) and Arctic amplification (*15*) in increasing early and mid-Holocene annual temperatures. However, these studies either fail to show an HTM and cooling thereafter in annual temperatures or exclude other important forcings that are known to affect Holocene temperatures, such as changes in GHGs.

One possible forcing mechanism that has not been thoroughly explored in modeling studies is the expansion of Holocene vegetation. Pollen records from the early and mid-Holocene show that grass and shrub vegetation expanded in the African Sahara (*16*, *17*), temperate deciduous forest cover increased in the NH mid-latitudes (*18*–*20*), and boreal forest replaced tundra in the Arctic (*21*–*23*). Increases in vegetation cover warm the land surface by enhancing the surface absorption of shortwave (SW) radiation directly through lowered albedo (*24*, *25*) and indirectly through limiting dust mobilization (*26*). While the impacts of NH vegetation change on regional Holocene climate, especially the African Sahara (*25*–*29*), have been widely studied, their effect on global annual temperatures is poorly understood. Furthermore, while some previous modeling studies have attempted to include the role of vegetation change with dynamic simulation of vegetation, they have failed to reproduce the vegetation extent inferred from pollen and, as a result, have likely underestimated vegetation-forced warming (*5*, *27*, *30*–*32*). In addition, some modeling studies have investigated regional climate responses to prescribed vegetation during the Holocene and in the future and find that increases in vegetation cover can produce warming at the regional scale (*24*, *33*, *34*).

In this study, we use the Community Earth System Model version 1.2 (CESM1.2) to investigate the impact that NH vegetation change in the Holocene has on global temperatures. We perform simulations for 9, 6, and 3 ka BP and the PI with varying prescriptions of vegetation in the African Sahara, NH mid-latitudes, and Arctic (table S1). In each Holocene experiment, we systematically increase vegetation cover from the PI extent to show the effect of regional vegetation change on regional and global surface temperatures (fig. S1). We then compare the results from our simulations with proxy reconstructions from T12K. Our results highlight the role of vegetation in driving past climate change.

## RESULTS

### Global temperature response to vegetation

**Fig. 1.**
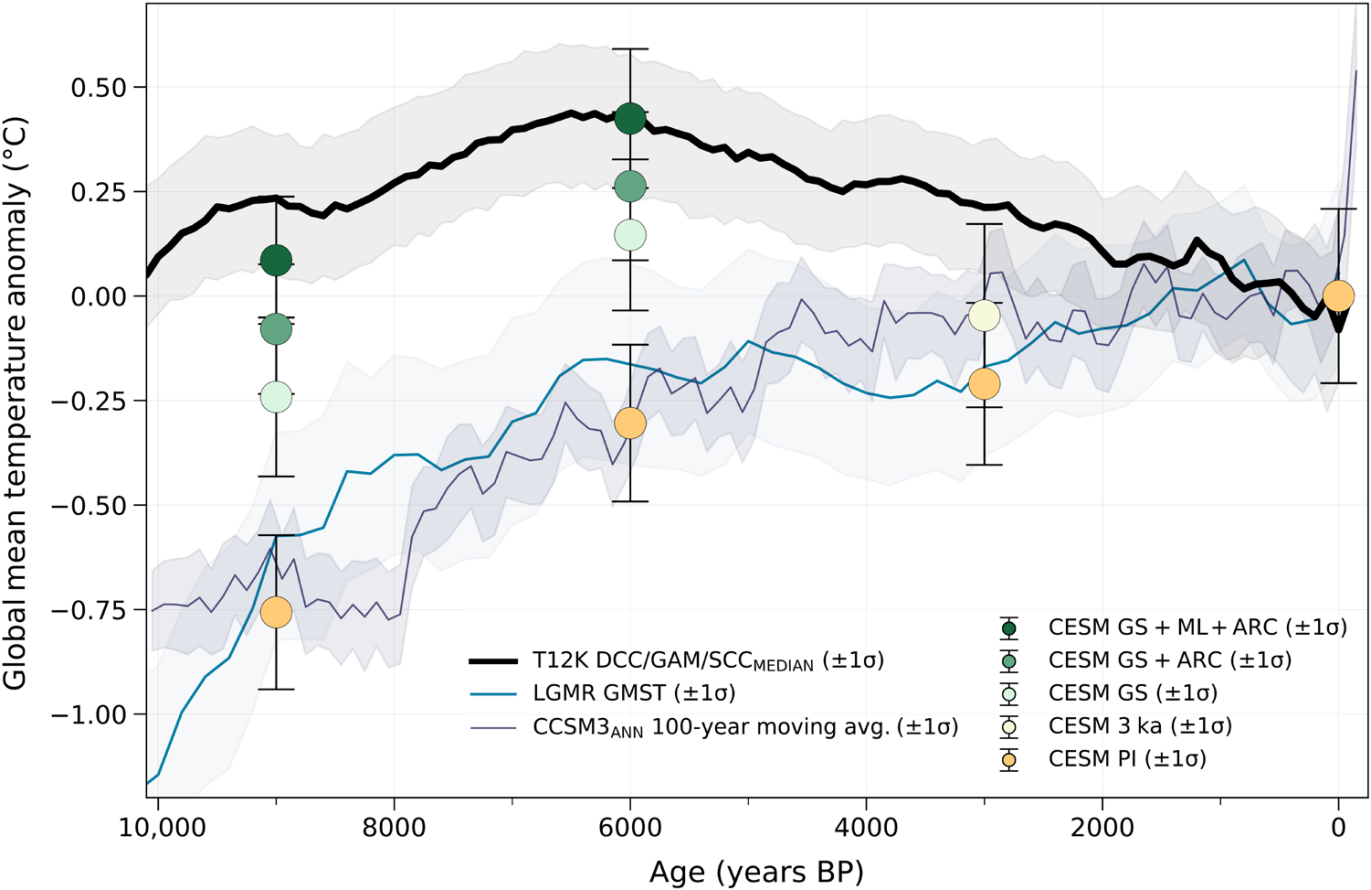
Model-proxy comparison of global mean surface temperature anomalies. Colored circles show CESM1.2 annual global mean surface temperature anomalies (±1 SD) for all simulations with various degrees of increased NH vegetation (GS, Green Sahara; ML, mid-latitude greening; ARC, Arctic greening; 3 ka, partial vegetation changes in the Sahara and NH mid-latitudes; PI, PI vegetational extent; see Materials and Methods and table S1). Our simulated temperature anomalies are compared with the median of both the global mean and SD from three T12K individual reconstructions (DCC, 782 records; GAM, 761 records; SCC, 761 records; black line, ±1 SD) (*4*), the Last Glacial Maximum reanalysis (LGMR) global mean surface temperature reconstruction (blue line, ±1 SD) (*12*), and the simulated global mean temperature anomalies from a transient CCSM3 simulation from the TraCE-21ka experiment (*47*, *48*) shown as the 100-year moving average (purple line, ±1 SD) from each 100-year interval. Time series anomalies are shown relative to the past 1 ka BP.

The incorporation of Holocene vegetation change in our simulations drives a mid-Holocene thermal maximum and produces a closer match to the global mean temperature anomalies in the T12K reconstruction (Fig. 1). Relative to simulations with PI vegetation (orange circles), simulated global temperatures increase by ~0.8° and 0.7°C at 9 and 6 ka BP (darkest green circles; 9ka and 6ka), respectively, with vegetation change over the African Sahara, NH mid-latitudes, and Arctic, and by ~0.2°C at 3 ka BP (tan circle; 3ka) with partial vegetation change over the Sahara and NH mid-latitudes. These increases in NH vegetation place our simulated temperature anomalies within the 1σ range of the median of T12K individual reconstructions (black line; see Materials and Methods).

Our Holocene simulations demonstrate that increased vegetation cover is a plausible mechanism for producing an HTM in climate model simulations and matching the T12K proxy reconstruction. Without regional vegetation change (orange circles; 9ka_PI_VEG_, 6ka_PI_VEG_, and 3ka_PI_VEG_), our simulated temperature anomalies are colder than the T12K curve by as much as ~1.0°C (Fig. 1), following the previous warming trends in TraCE-21ka and Paleoclimate Model Intercomparison simulations (*35*), as well as the recent Last Glacial Maximum reanalysis (LGMR) (*12*). Furthermore, the timing and magnitude of the HTM in our annual temperature anomalies correspond well with the T12K curve, suggesting that the ~8 to 6 ka BP peak could be attributed to a waning ice sheet and vegetation change. In comparing proxy reconstructions with our boreal summer [June, July, and August (JJA)] global mean temperature anomalies, we find that our simulations no longer match T12K and instead shift the timing of the HTM to 9 ka BP (fig. S2), in line with NH JJA insolation (*8*). JJA temperature anomalies in TraCE-21ka simulations, where dynamical simulation of vegetation did not adequately produce changes inferred from pollen records, remain colder than in T12K, especially at 6 ka BP (fig. S2, blue line). This further suggests that vegetation, rather than boreal summer insolation, produces a mid-Holocene HTM.

### Regional warming by vegetation

**Fig. 2.**
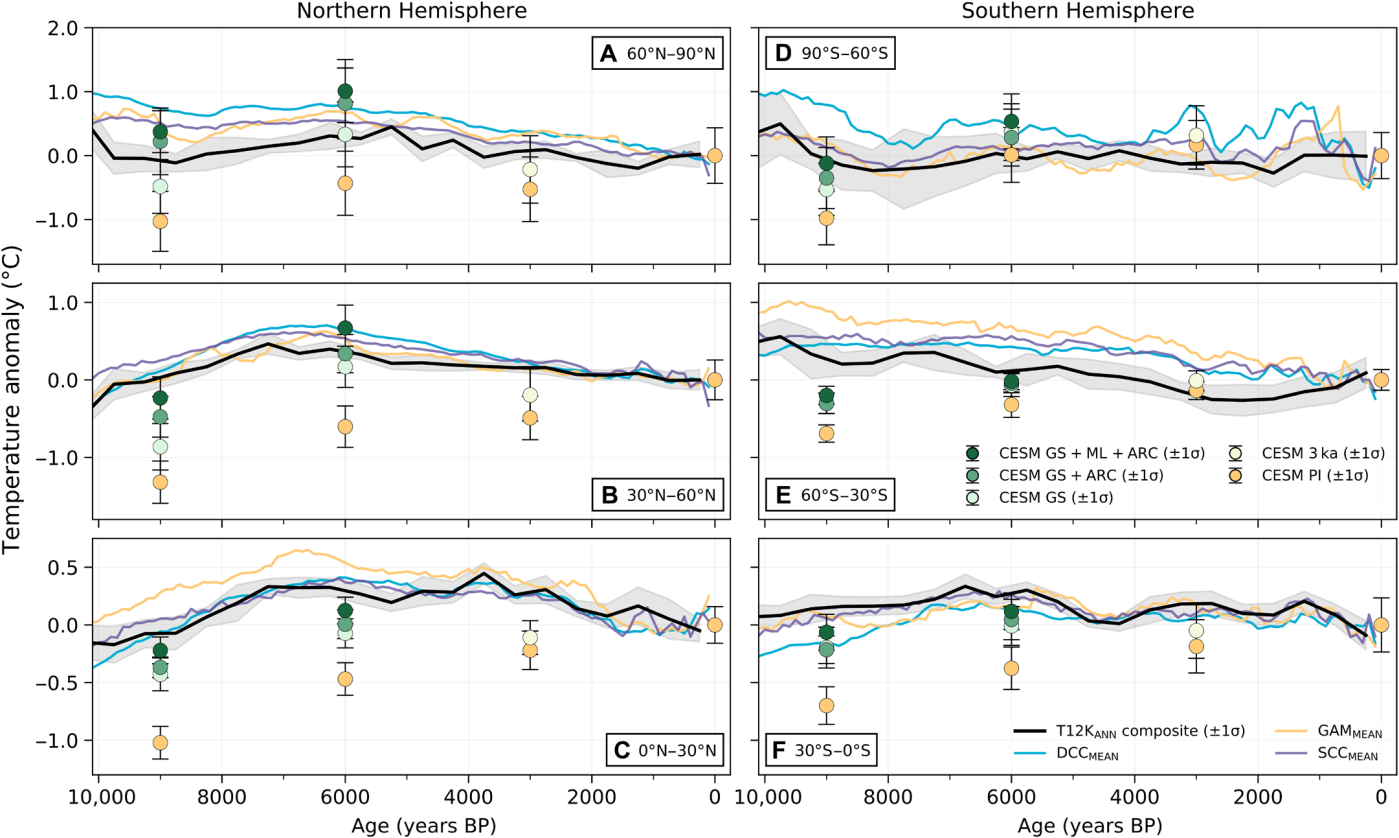
Model-proxy comparison of zonally averaged surface temperature anomalies. Colored circles show zonally averaged annual CESM1.2 surface temperature anomalies (±1 SD) calculated only from model grid cells corresponding to the T12K_ANN_ composite. Simulated temperature anomalies are compared at each 30° latitude band with the T12K_ANN_ composite (black line, ±1 SD) (*3*) and individual statistical reconstructions of mean surface temperature anomalies from T12K (DCC, blue line; GAM, orange line; SCC, purple line) (*4*). Note the differences in *y*-axis values between latitude bands. Time series anomalies are shown relative to the past 1 ka BP. Numbers of proxy records from T12K_ANN_ in each latitude band are (**A**) 71, (**B**) 295, (**C**) 89, (**D**) 11, (**E**) 31, and (**F**) 83.

**Fig. 3.**
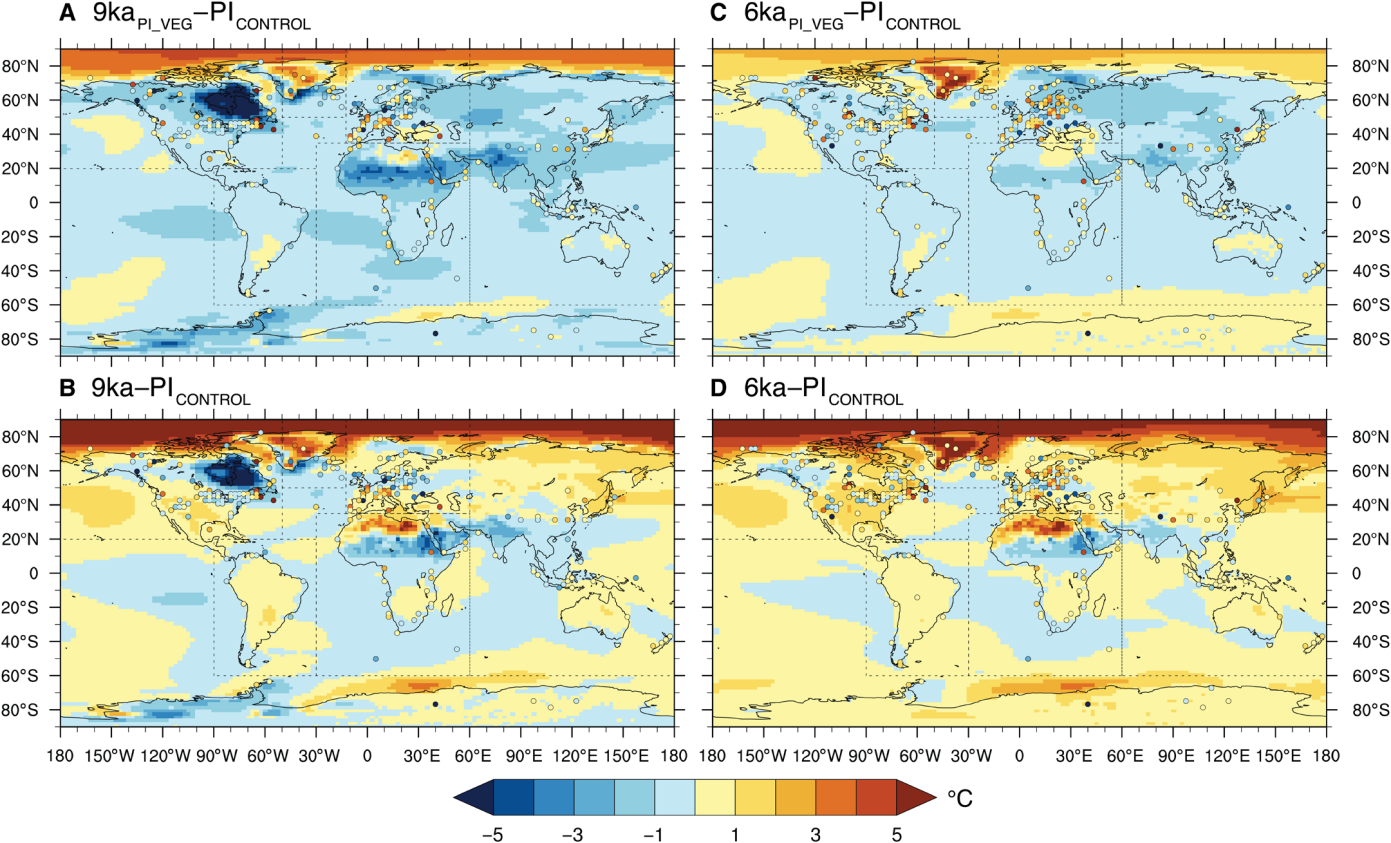
Global comparison of annual Δ*T* between CESM1.2 and the T12K_ANN_ composite. The colors of dots (shading) correspond to (**A**) 9 ka BP–PI (9ka_PI_VEG_–PI_CONTROL_), (**B**) 9 ka BP–PI (9ka–PI_CONTROL_), (**C**) 6 ka BP–PI (6ka_PI_VEG_–PI_CONTROL_), and (**D**) 6 ka BP–PI (6ka–PI_CONTROL_). Regions defined for spatial analysis are shown by black dashed lines. The lone record in the Atlantic Ocean at ~40°N and ~30°W is included with proxies from Europe.

The incorporation of vegetation change in our Holocene simulations also largely resolves the model-data discrepancy in zonally distributed regional temperature inferred from T12K (Fig. 2). Since Holocene temperature anomalies vary regionally in proxy reconstructions, with the largest warming taking place in the high latitudes and lessening toward the equator (*3*–*5*, *12*), any mechanism (i.e., vegetation cover) used to explain the Holocene Temperature Conundrum must also account for this heterogeneity in temperature anomalies. Hence, we compare our model results with T12K proxies, from both annual composites (hereafter referred to as T12K_ANN_) and individual reconstructions of mean surface temperature (see Materials and Methods), across six zonal bands (Fig. 2). Our simulations with PI vegetation (orange circles) fail to capture the reconstructed anomalies at all latitude bands and are, on average, colder than the T12K_ANN_ composite anomalies at 9, 6, and 3 ka BP by 1.0°, 0.6°, and 0.3°C, respectively. We note that the high latitude temperature response in our 6ka_PI_VEG_ simulation (Fig. 3) differs from that in (*15*), which used the same climate model but only incorporated changes in orbital forcing. In comparison, our simulation had additional forcing from lower, more realistic GHG concentrations [e.g., 264.4 parts per million (ppm) CO_2_ in our study versus 284.7 ppm CO_2_ in (*15*)]. The colder temperature anomalies in our simulation are in line with the latest paleoclimate modelling intercomparison project phase 4 (PMIP4) multimodel ensembles that had similarly lower GHGs, relative to PMIP3 (*35*).

The simulations with specified vegetation change simulate warmer regional temperatures north of 30°S and more closely match T12K (*3*, *4*). North of 30°N at 9 and 6 ka BP, some of our vegetated simulations do exhibit higher temperature anomalies than T12K_ANN_; however, their anomalies still fall within the spread from the T12K individual reconstructions. At these latitudes, these vegetated simulations still align more closely with the proxy reconstructions as a whole than do the simulations with PI vegetation (Fig. 2, A and B). South of 30°S, low data density (31 or fewer records in 30°S to 60°S and 11 records in 60°S to 90°S) likely increases the uncertainty in model-data comparison and precludes a robust assessment for these regions, although the simulations with vegetation change appear to match the data better at 9 ka BP (Fig. 2, D and E).

**Fig. 4.**
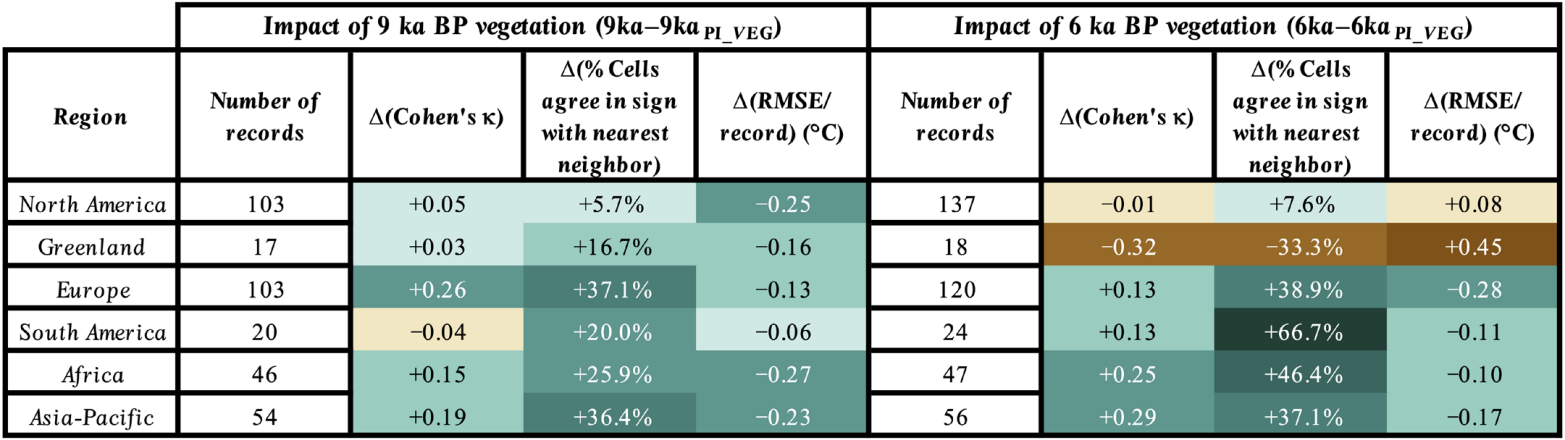
Regional improvement in model-data agreement by NH vegetation change. Comparison between CESM1.2 and T12K_ANN_ composite shown as increased agreement (green) and decreased agreement (brown), shaded by degree of change. Detailed description of each method can be found in Materials and Methods and Supplementary Text. Full statistical results for all 9 and 6 ka BP simulations are included in data file S1.

Since 9 and 6 ka BP experience large vegetation-induced warming in our simulations, we evaluate the spatial agreement in annual temperature between our simulations and the T12K_ANN_ composite at these two time slices for six regions: North America, Greenland, Europe, South America, Africa, and Asia-Pacific (Fig. 4). These regions include all annual composite records north of 60°S in a compilation encompassing both terrestrial and marine records (Fig. 3). The few open ocean records are grouped with the closest geographical terrestrial and ocean margin records. Antarctica is excluded from this analysis as low data density below 60°S inhibits robust conclusions for this region.

For our spatial analysis, we compare the temperature difference (Δ*T*) between 9 or 6 ka BP and the PI between T12K_ANN_ and our simulations. To illustrate the improvement in model-data agreement due to NH vegetation change, we quantify the difference in model-data agreement between simulations with NH vegetation change (e.g., 9ka–PI_CONTROL_) and PI vegetation (e.g., 9ka_PI_VEG_–PI_CONTROL_) (see Materials and Methods). To quantitatively assess the improvements in model-data agreement, we calculate the differences in weighted Cohen’s κ statistic, the percentage of nearest-neighbor model grid cells that agree in sign of Δ*T*, and root mean square error (RMSE) normalized by the number of proxy records present in each region (see Materials and Methods).

We find better model-data agreement in ~81% of categories across the six regions due to NH vegetation change in our simulations (Fig. 4). The strongest improvements occur in Asia-Pacific, Africa, and Europe, regions directly affected by the prescribed vegetation expansion, and in South America, a region with known teleconnections to the African land surface (*36*). In these regions, on average, the weighted Cohen’s κ statistic increases by 0.17, the nearest-neighbor grid cells that agree in sign increase by 38.6%, and RMSE decreases by 0.17°C. In North America, where prescribed vegetation change also occurs, modest improvements in these categories may result from uncertainties relating to the Laurentide Ice Sheet at 9 ka BP and enhanced warming north of 70°N where few proxy records exist. Mixed improvement in model-data agreement due to vegetation change occurs in Greenland, likely as a result of low proxy data density and Greenland’s distance from prescribed vegetation change (*37*).

### Global temperature response to dust

**Fig. 5.**
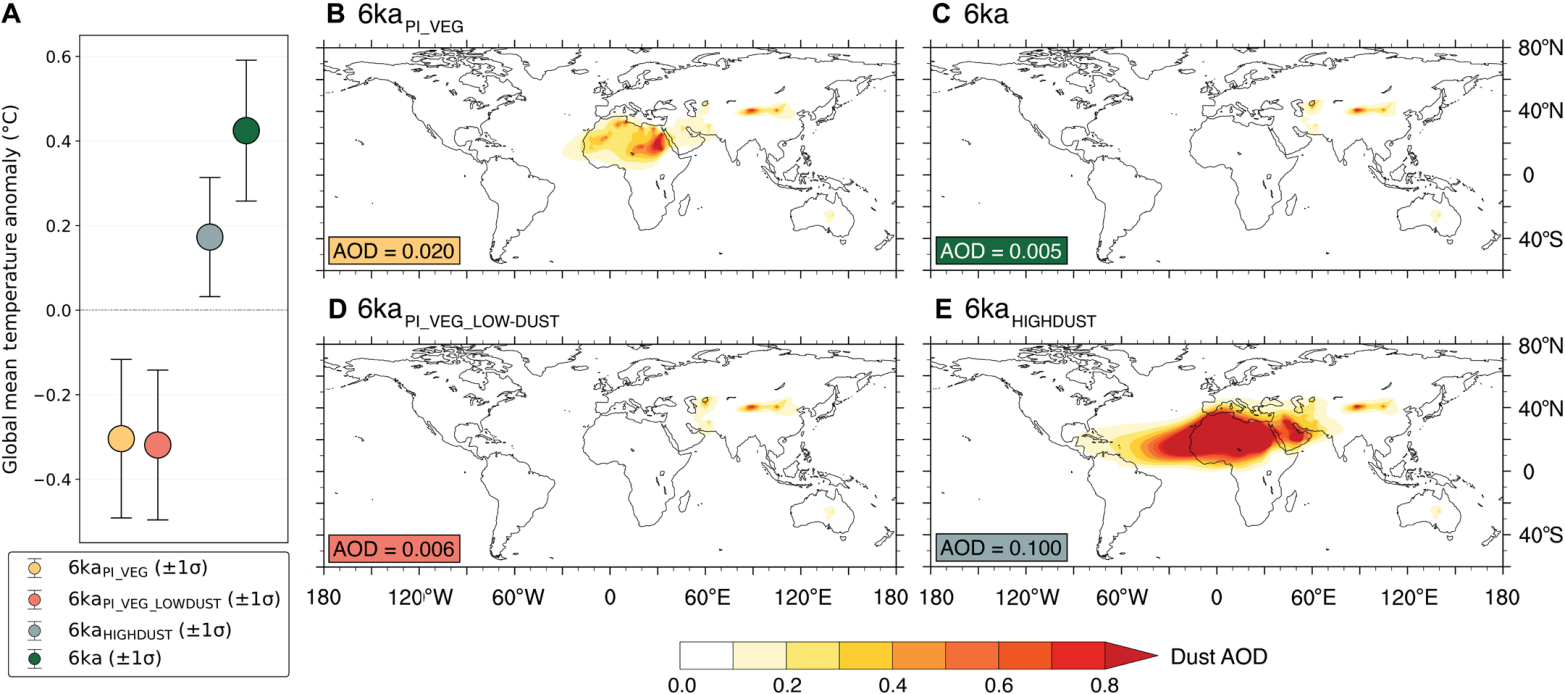
Global temperature change as a result of dust during 6 ka BP. (**A**) Annual global mean surface temperature anomalies (relative to PI_CONTROL_) for 6ka_PI_VEG_ (far left), 6ka_PI_VEG_LOWDUST_ (middle left), 6ka_HIGHDUST_ (middle right), and 6ka (far right). Annual dust AOD shown in (**B**) to (**E**). Global mean dust AOD for each simulation is reported in the bottom left of each panel with its color corresponding to (A).

Increased NH vegetation cover also resulted in reduced dust aerosol loading in 9 and 6 ka BP (*38*, *39*), which itself has been suggested as a potential solution to the Holocene Temperature Conundrum (*14*). To investigate vegetation-induced dust forcing at 6 ka BP, where we see the largest simulated anomalous warming, we run two additional sensitivity experiments that highlight the global temperature response to dust reduction with and without NH vegetation change (see Materials and Methods). To test the response without NH vegetation change, we first run a simulation referred to as “6ka_PI_VEG_LOWDUST_” that is identical to 6ka_PI_VEG_ but has 72% lower dust aerosol optical depth (AOD) (table S1). In this sensitivity experiment, reduced dust does not lead to an increase in the global temperature anomaly (Fig. 5). Furthermore, to test the global temperature response to a hypothetical dust reduction with NH vegetation change, we run an additional simulation referred to as “6ka_HIGHDUST_” that is identical to 6ka (with NH vegetation expansion) but has extremely high dust AOD (~400% higher than 6ka_PI_VEG_; dust AOD of ~0.1 versus ~0.005 in 6ka; table S1). The hypothetical high dust loading in 6ka_HIGHDUST_ decreases the global temperature anomaly by ~0.25°C (Fig. 5), compared to 6ka. This result demonstrates that the total warming due to the combined effect of increased vegetation and reduced dust at 6 ka BP (6ka–6ka_PI_VEG_), which is approximately 0.73°C, is primarily the result of direct vegetation change because an extreme reduction in dust can, at most, explain ~35% of this warming.

### Mechanism for warming

In accordance with previous studies (*24*, *40*, *41*), we find that vegetation-induced changes in surface albedo largely drive the increase in regional surface temperatures. We diagnose the SW radiative effect of NH vegetation change in 6 ka BP sensitivity experiments using approximate partial radiative perturbation (APRP) analysis (see Materials and Methods) (*42*). We find that the SW radiative forcing associated with changes in vegetation albedo is +2.41 W/m^2^ (table S2), of which +1.91 W/m^2^ comes from greening of the African Sahara where the shift from bare ground (desert) in the PI to shrub and grassland in 6 ka BP decreases albedo in our model by as much as 0.29 (*43*). The change in SW forcing by albedo outweighs modest decreases in global radiative feedbacks from clouds and noncloud constituents (i.e., aerosols). Greening of the African Sahara leads to an increase in the net surface radiative flux of +15 W/m^2^ and contributes to surface warming at all latitudes (fig. S3). Warming is particularly large in the Arctic, where NH vegetation amplifies warming initially driven primarily by sea ice loss (*15*).

## DISCUSSION

### Potential biases in model-proxy comparison

Although T12K is the most complete global proxy compilation to date, it still may be subject to spatial bias since most of the available temperature proxies in T12K come from terrestrial sites in the NH mid-latitudes (*3*, *4*). Only ~16% of T12K records are from the Southern Hemisphere, and ~6% are from the high (>70°N) northern latitudes. The lack of high-latitude records may partly explain why our simulated Arctic temperatures are warmer than T12K composites (Figs. 2 and 3 and fig. S4). Some proxy data suggest that early to mid-Holocene annual temperatures in Greenland may have been as much as 1° to 2°C higher than at present, in agreement with our simulated high latitude temperatures (*44*–*46*). Furthermore, we find that our simulated global mean temperature anomalies at 6 ka BP are ~0.2°C higher than when the global temperature is calculated using only model grid cells at the T12K_ANN_ sites (comparison between Fig. 1 and fig. S5). This suggests that the uneven spatial distribution of T12K_ANN_ sites underestimates the “true” HTM by a small amount (~0.2°C) if a simple area-weighted mean is used (see Materials and Methods).

Other potential biases may exist in the sensitivity of our model, CESM1.2, to the degree of our prescribed changes in NH vegetation. Our prescribed NH vegetation change is larger than previous studies (*5*, *47*, *48*) and so may partially contribute to the simulated large warming. For instance, we follow PMIP4 Tier 2 protocol that calls for 100% vegetation cover in the African Sahara (*49*) as opposed to a mixed environment with shrubs, grasses, and bare ground (*16*, *17*). As previous work has shown, differences in plant type and diversity when simulating the Green Sahara can affect the resulting climate (*50*). Therefore, our simulations may represent the high end of potential Holocene vegetation change and the resulting temperature response. However, our choices of prescribed vegetation are rooted in evidence from several pollen studies (*9*, *16*, *17*, *22*, *23*) and highlight the substantial ways in which changes in vegetation affected Holocene climate.

One potential limitation of our study is that our conclusions are based on a single climate model (CESM1.2). Our choice in using CESM1.2 is based on its widely acknowledged skill in the simulation of both past and present climates (*51*–*54*). We believe that our results from CESM1.2 are likely representative of other models, given that the vegetation-induced surface warming relies on simple radiation processes and is largely independent of model physical parameterizations (*24*, *33*, *55*, *56*). Nonetheless, we encourage future work to leverage model ensembles with updated dynamic vegetation schemes to further refine Holocene estimates of NH vegetation change and its subsequent impact on global and regional temperatures.

### Reconstruction of the HTM

Our results show that vegetation-induced warming gives rise to an HTM at 6 ka BP, followed by a cooling trend toward the PI. In contrast, our simulations that do not include the NH vegetation expansion, but only account for changes in orbital insolation, GHGs, and ice cover, predict long-term warming through the Holocene.

Our findings differ from those of recent studies that suggest that seasonal biasing in proxy records was the source of the HTM (*13*, *57*). We attribute this disparity to the location of their records and their SST-only approach. These recent studies interpret their reconstructions as being indicative of global temperature, despite the fact that they represent tropical and subtropical SSTs, which have been shown to exhibit a muted HTM signal when compared to air temperatures from higher latitudes (*58*). To test the assumption that their records represent global temperatures, we estimate the average 6 ka BP SST using only model grid cells corresponding to the tropical locations from (*13*). We find that this average SST is ~0.25°C colder than the global mean air temperature at 6 ka BP (fig. S6), implying that the proxy reconstructions are not representative of global mean air temperatures. Whether the other records from T12K, most of which are terrestrial pollen records, exhibit a seasonal bias that is not accounted for in the reconstruction technique remains an open question (*5*, *13*), yet one recent study of European pollen records suggests that they do represent annual temperature (*11*).

In addition, the recent data assimilation of surface temperature since the Last Glacial Maximum (the LGMR), which integrates global SST proxies with a subset of the model simulations used in this study (see Materials and Methods), finds that seasonal bias has a relatively minor influence on SST-based reconstructions (*12*). However, the LGMR, unlike proxy-only reconstructions, does not show a distinct HTM, although it does show more early Holocene warmth than TraCE-21ka (Fig. 1, blue versus purple line). The LGMR includes simulations with a varying degree of NH vegetation change in its prior state; hence, the cooler mid-Holocene may reflect a preference from the proxies (*12*). However, the influence of the absence of terrestrial temperature data in the LGMR, and whether it might lead to a more pronounced mid-Holocene warming, is unknown and requires further investigation (*59*).

In summary, we show that NH vegetation change can drive mid-Holocene warming in annual global mean air temperatures. This warming closely aligns our model simulations with temperature anomalies from T12K, providing a potential solution to the Holocene temperature conundrum between proxy data and climate models. Our results demonstrate that vegetation is an important driver of temperature change during the Holocene, and other mechanisms, such as dust, ice cover, orbital forcing, or GHGs cannot produce early and mid-Holocene warmth without the changes in NH vegetation. Our findings further highlight the substantial influence of vegetation expansion and contraction on global climate. These results demonstrate that IPCC-class models (e.g., CESM1.2) can simulate a realistic temperature response to external climate forcings but only when all relevant forcings are included. Our findings imply that future climate projections that include changes in vegetation are likely to produce more trustworthy predictions of future climate change.

## MATERIALS AND METHODS

### Climate model simulations

We ran the fully coupled CESM1.2 at 3000-year intervals between 9 ka BP and the PI. The full list of simulations and respective parameters, boundary conditions, and variables of interest can be found in table S1. CESM1.2 is composed of the Community Atmosphere Model version 5.3, Community Land Model 4.0, Community Ice Code version 4.0, Parallel Ocean Program version 2, River Transport Model, and a coupler connecting them (*60*). The atmosphere model, with 30 vertical levels, and the land model, with 15 soil-column layers (*43*), were run with a grid resolution of 1.9° × 2.5°, while the ocean model was run with nominal 1° resolution. Each simulation contained orbital forcing, GHG concentrations (CO_2_, CH_4_, and N_2_O), and ice sheet reconstruction from the ICE-6G (*61*), consistent with its respective time period. All simulations were run until the top-of-atmosphere energy imbalance was less than 0.1 W/m^2^. When calculating annual mean temperature, we adjusted values and weighted by the change in fraction of each month to account for the paleo calendar effect (*62*). Climatologies were calculated from the last 50 years of each simulation.

To isolate the impact of NH regional vegetation change, we performed sensitivity experiments for 9, 6, and 3 ka BP (table S1). Each of these simulations contained climate parameters (orbit year, GHGs, and ice cover) for their respective time period, and these were compared to both a preindustrial control simulation entitled “PI_CONTROL_” and a simulation for each time period containing prescribed vegetation consistent with the PI, entitled “9ka_PI_VEG_,” “6ka_PI_VEG_,” and “3ka_PI_VEG_” (orange boxes in fig. S1 and orange circles in Fig. 1). Details of the PI_CONTROL_ and 3ka_PI_VEG_ simulations can be found in the works of Tierney *et al.* (*63*) and Zhu and Poulsen (*64*). A subset of the model simulations used in this study (PI_CONTROL_, 3ka, 6ka_PI_VEG_, 6ka_GS + ARC_, and 9ka_GS_) were used as model priors in the work of Osman *et al.* (*12*). Vegetation phenology was prescribed in accordance with satellite observations, demonstrating a best estimate for PI vegetation. Subsequent variations in vegetation change were performed for 9, 6, and 3 ka BP in the African Sahara, NH mid-latitudes, and Arctic (fig. S7).

### Prescribed vegetation

For both 9 and 6 ka BP, vegetation was incrementally increased in the African Sahara, NH mid-latitudes, and Arctic in accordance with PMIP4 Tier 2 guidelines (*49*) and evidence from pollen records (*9*, *16*, *17*, *22*, *23*). We accounted for the Green Sahara, which expanded grass and shrub vegetation and enhanced the African hydrologic cycle (*16*, *17*, *26*–*28*), in a sensitivity experiment entitled “9ka_GS_” and “6ka_GS_” for 9 and 6 ka BP. This experiment greened the African Sahara by replacing bare ground desert with 100% shrub at ~10°N to 25°N and 100% C_4_ grass at 25°N to 35°N (fig. S7A; light green boxes in fig. S1 and light green circles in Fig. 1). Since vegetation replaced bare ground in these simulations, we prescribed leaf area index with summer values reaching as high as 3.0 for shrublands and 1.5 for grasslands. We accounted for increases in Arctic rainfall and plant available moisture that led to replacement of tundra by boreal forest (*9*, *18*, *20*–*23*) with another sensitivity experiment entitled “9ka_GS + ARC_” and “6 ka_GS + ARC_.” This experiment added greening of the Arctic, through replacement of all C_3_ grass north of 50°N with boreal forest, to the previously mentioned Saharan greening (fig. S7B; green boxes in fig. S1 and green circles in Fig. 1). Last, we accounted for increases in NH mid-latitude rainfall and plant available moisture that expanded temperate deciduous forest cover (*9*, *18*–*20*) with a sensitivity experiment entitled “9ka” and “6ka.” This experiment added NH mid-latitude greening, through replacement of C_3_ grass between 30°N and 60°N with deciduous forest, to the previously mentioned Saharan and Arctic greening (fig. S7C; dark green boxes in fig. S1 and dark green circles in Fig. 1).

At 3 ka BP, pollen records (*21*, *65*) suggest a slight increase in moisture that may have shifted the boundary between the African Sahel and Sahara north by ~3° to 5° of latitude (*66*), while the extent of temperate deciduous forest expanded (*18*). A sensitivity experiment entitled “3ka” accounted for these changes by shifting the Sahara/Sahel boundary north by 5° of latitude through prescription of shrub from ~10°N to 16°N and replacing 50% of C_3_ grassland between 40°N and 60°N with deciduous forest (fig. S7D; tan box in fig. S1 and tan circle in Fig. 1).

### Dust experiments

Two additional sensitivity experiments were performed at 6 ka BP to isolate the impact of reduced dust emissions with and without NH vegetation change (Fig. 5). The simulation entitled 6ka_PI_VEG_LOWDUST_ contained a PI vegetational extent with dust emissions lowered to 6 ka BP levels, in accordance with reconstructions (*38*, *39*, *67*, *68*), and the simulation entitled 6ka_HIGHDUST_ contained identical prescribed NH vegetation as 6ka but allowed for dust mobilization as if the vegetation were the PI extent (table S1). While 6ka_HIGHDUST_ increased dust AOD by nearly 400% compared to 6ka_PI_VEG_, it enabled us to calculate an extreme high endmember contribution of reduced dust to global temperature change. This was not meant to be a realistic simulation but rather provided for quantification of the maximum possible contribution of dust in increasing global temperatures. In CESM1.2, the dust model prohibits dust from mobilizing when leaf area index exceeds 0.3 (*43*); however, these two 6 ka BP sensitivity experiments were modified to not follow this rule. 6ka_PI_VEG_LOWDUST_ did not mobilize dust even in areas with leaf area index of less than 0.3, and 6ka_HIGHDUST_ mobilized dust in areas where leaf area index exceeded 0.3. All other 9 and 6 ka BP experiments that included increased NH vegetation followed this rule and, as a result, had greatly reduced dust aerosol loading relative to PI_CONTROL_ (table S1).

### APRP feedback analysis

To calculate the SW radiative effects of NH vegetation change at 6 ka BP, we used the APRP method (*42*). This method isolated the top-of-atmosphere SW radiative responses of surface albedo, cloud, and noncloud atmospheric constituents to vegetation change in 6 ka BP sensitivity experiments relative to 6ka_PI_VEG_ (table S2). SW forcing has been shown to play the predominant role in vegetation-induced warming (*24*, *33*, *43*, *69*), so we neglected longwave forcing, which requires more extensive calculation (*70*). For this analysis, we used a climatology of the last 100 years of model output for the 6 ka BP sensitivity experiments and the last 50 years for 6ka_PI_VEG_.

### T12K temperature proxy composites

We used temperature proxy records from the T12K database (*71*) to compare with our CESM1.2 simulations. All proxy and model time series reconstructions shown in this study are presented as temperature anomalies relative to the average of their last 1000 years (1 to 0 ka BP), while CESM1.2 temperature anomalies are relative to PI_CONTROL_. For our global model-data comparison (Fig. 1), we compared our simulated annual global mean surface temperature anomalies against the median value of the global mean temperature anomalies and their SDs over the Holocene from three T12K statistical reconstructions [dynamic calibrated composite (DCC), general additive model (GAM), standard calibrated composite (SCC)] (*4*). Each of these three reconstructions used a different statistical approach to aggregate annual and seasonally biased T12K proxies from 679 sites into a single binned time series of global temperature anomalies that included 500 ensemble members each. We excluded pairwise comparison (PAI) and composite plus scale (CPS) from our analysis because these reconstructions are relative outliers in their reconstruction of both global and Arctic temperatures. However, since we use the median value of the individual reconstructions, this choice does not greatly affect the resulting global T12K temperature reconstruction shown in Fig. 1 (black line). We calculated our global average temperature anomalies by weighting each 30° zonal value by its proportion of global area in accordance with (*4*). In our regional comparison (Fig. 2), we showed the three statistical reconstructions (DCC, GAM, and SCC) individually and also compared against a composite of 580 publicly available annually calibrated records from the T12K database (referred to in this study as T12K_ANN_) (*3*, *4*). To compare directly between our modeling results and proxy records, we calculated zonal mean surface temperatures from CESM1.2 by only including model grid cells corresponding to the T12K_ANN_ composite.

We highlighted the time evolution of the median value from T12K individual reconstructions and the simulated global mean temperature anomalies from CESM1.2 to show as close to a global like-for-like comparison as possible in Fig. 1. When instead calculating the CESM1.2 mean from only model grid cells associated with the annually calibrated proxy reconstructed temperatures from the T12K_ANN_ composite, our simulated temperature anomalies at the HTM were ~0.2°C colder (compare Fig. 1 to fig. S5), showcasing that the uneven sampling distribution of T12K_ANN_ records likely leads to a cold bias in global temperature.

For our spatial comparisons of North America, Greenland, Europe, South America, Africa, and Asia-Pacific, we calculated T12K_ANN_ proxy Δ*T* as 9 ka BP–PI and 6 ka BP–PI by averaging proxy values between 9.5 and 8.5 ka BP or 6.5 and 5.5 ka BP and subtracting from them the PI average of 0.5 ka BP–present. When more than one proxy record was located spatially within the same model grid cell, we averaged the proxy Δ*T* values to produce a single Δ*T* value for that grid cell.

### Quantification of improvements in model-data agreement

We used three distinct methods to quantify the improvement in model-data agreement due to increased NH vegetation at 9 and 6 ka BP (see Supplementary Text for more detailed explanations of each method). First, we assessed the change in the weighted Cohen’s κ statistic (*72*, *73*) as a result of increased vegetation. This method has been shown to be a robust measure of model-data agreement (*74*), and we use it here to quantify how well the model and data results agree in sign of Δ*T*, relative to their agreement by random chance, by using two distinct categories: “warmer” and “colder.” If the model and data are in complete agreement, then κ = 1, and if there is no agreement between the model and data other than is expected by chance, then κ = 0. Positive values in Fig. 4 indicate higher Cohen’s κ values as a result of NH vegetation change and improved model-data agreement.

Second, we calculated the percentage of nearest-neighbor grid cells where both the model and proxy record agreed in sign of Δ*T* (fig. S8). This method controls for uncertainty in the spatial locations of proxies and their corresponding grid cells in the model by allowing positive matches to occur in adjacent grid cells.

Third, we calculated the change in RMSE normalized by the regional numbers of proxy records, which effectively measured the average difference in Δ*T* (°C) between each proxy record and its corresponding grid cell in the model. This method is useful for understanding the agreement in overall magnitude of change in model and proxy Δ*T*.

While inherent uncertainties may exist within the calculation of any one of these methods, analysis of them as a whole provides valuable insight into the regional improvements in model-data agreement brought about by increased NH vegetation and accounts for uncertainties relating to agreement by random chance, alignment of spatial locations in models and proxies, and the overall magnitude of change in Δ*T*.
