# Supplementary material for: Northern Hemisphere vegetation change drives a Holocene thermal maximum
Source: Sci Adv. 2022 Apr 15;8(15):eabj6535. doi: 10.1126/sciadv.abj6535 (PMC9012463; doi:10.1126/sciadv.abj6535)
Supplement: Supplementary file 1 — Supplementary Text Figs. S1 to S8 Tables S1 and S2 [file sciadv.abj6535_sm.pdf]

Science Advances
AAAS

Supplementary Materials for

Northern Hemisphere vegetation change drives a Holocene thermal maximum

Alexander J. Thompson*, Jiang Zhu, Christopher J. Poulsen,
Jessica E. Tierney, Christopher B. Skinner

*Corresponding author. Email: alexjt@umich.edu

Published 15 April 2022, *Sci. Adv.* **8**, eabj6535 (2022)
DOI: 10.1126/sciadv.abj6535

The PDF file includes:

Supplementary Text
Figs. S1 to S8
Tables S1 and S2
Legend for data file S1

Other Supplementary Material for this manuscript includes the following:

Data file S1

Supplementary Text

We utilized three distinct methods for quantifying the improvement in model-data agreement as a result of NH vegetation change at 9 and 6 ka BP: weighted Cohen's κ statistic, the percentage of nearest-neighbor grid cells where both the model and proxy record agree in sign of ΔT, and root-mean-square error (RMSE) normalized by the regional number of proxy records. Full results of our statistical analyses for all three methods can be found in Data File S1.

To calculate the weighted Cohen's κ statistic for each region, we quantified agreement between the CESM1.2 and $T12K_{ANN}$ using the following categories:

		CESM1.2	
		warmer	colder
$T12K_{ANN}$	warmer	a	c
	colder	b	d

The value of κ was determined by the following equation:

$$\kappa = \frac{p_o - p_e}{1 - p_e}$$

where p_o is defined as the agreement between models and proxies, calculated by:

$$p_o = \frac{a + d}{N}$$

where N is the sum of all elements in each category (i.e., a to d) and p_e is the probability that the models and proxies agree by chance, calculated by:

$$p_e = \left[\frac{a + c}{N} * \frac{a + b}{N}\right] + \left[\frac{b + d}{N} * \frac{c + d}{N}\right]$$

We weight the κ value to penalize for total misses (e.g., if warmer should be colder) by multiplying the matrix above by the following weight matrix:

1	0
0	1

To determine the percentage of nearest-neighbor grid cells where both the model and proxy record agree in sign of ΔT, we defined two categories of model-data agreement ("warmer" or "colder") and calculated the total number of grid cells that exhibit model-data agreement with at least one adjacent model grid cell. Values for all 9 and 6 ka BP simulations can be found in Data File S1. An example of adjacent cell agreement is shown in Figure S8.

To determine RMSE normalized by the regional number of proxy records, we first calculated the difference (CESM1.2–$T12K_{ANN}$) at each grid cell. The sum of this difference across

all regional grid cells was then divided by the total number of proxy records present within the region. Results for all simulations can be found in Data File S1.

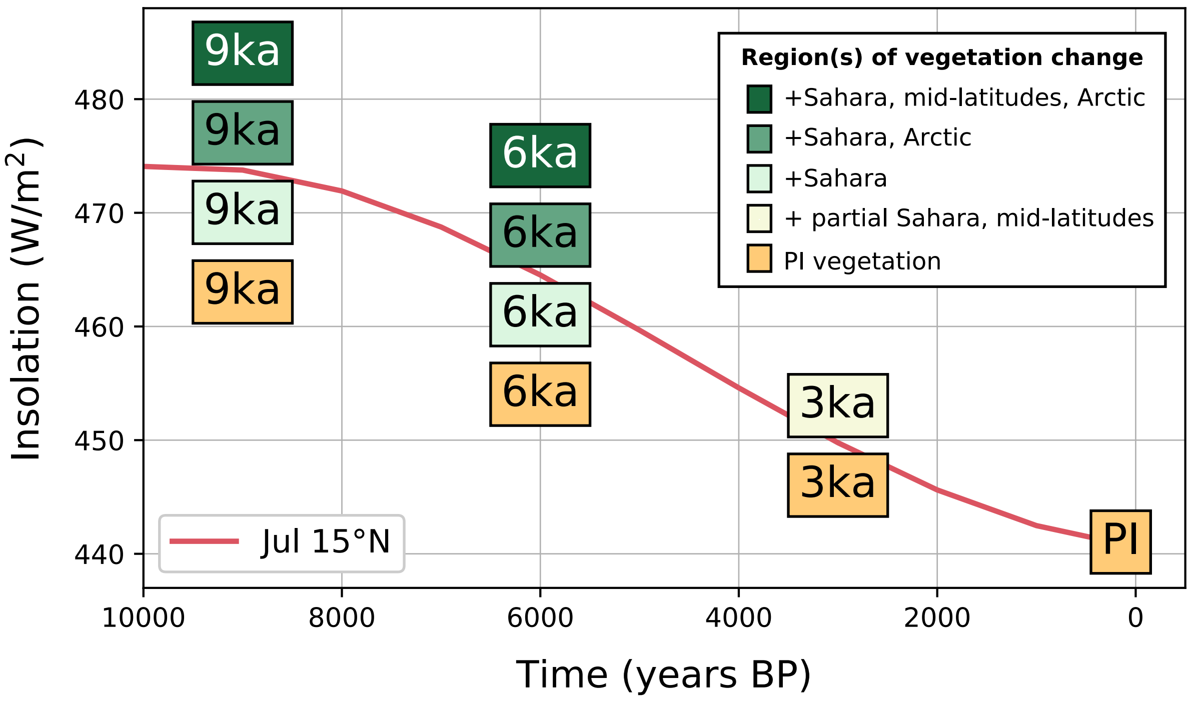

Figure S1. Suite of CESM1.2 simulations and respective vegetation modifications performed at each orbit year. Colors represent specified vegetation for each simulation: PI vegetation (orange; used in simulations $PI_{CONTROL}$ and $*_{PI\_VEG}$), 3 ka BP partial Saharan and mid-latitude greening (tan; used in *3ka*), greening of the Sahara (light green; used in $9ka_{GS}$ and $6ka_{GS}$), both Saharan and Arctic greening (green; used in $9ka_{GS+ARC}$ and $6ka_{GS+ARC}$), and Saharan, Northern Hemisphere mid-latitude, and Arctic greening (darkest green; used in *9ka* and *6ka*). See Table S1 for details on prescribed vegetation in the Sahara, Northern Hemisphere mid-latitudes, and Arctic and Fig. S7 for details regarding the spatial modification of plant functional types. July insolation at 15°N (red line) is shown for reference (*8*).

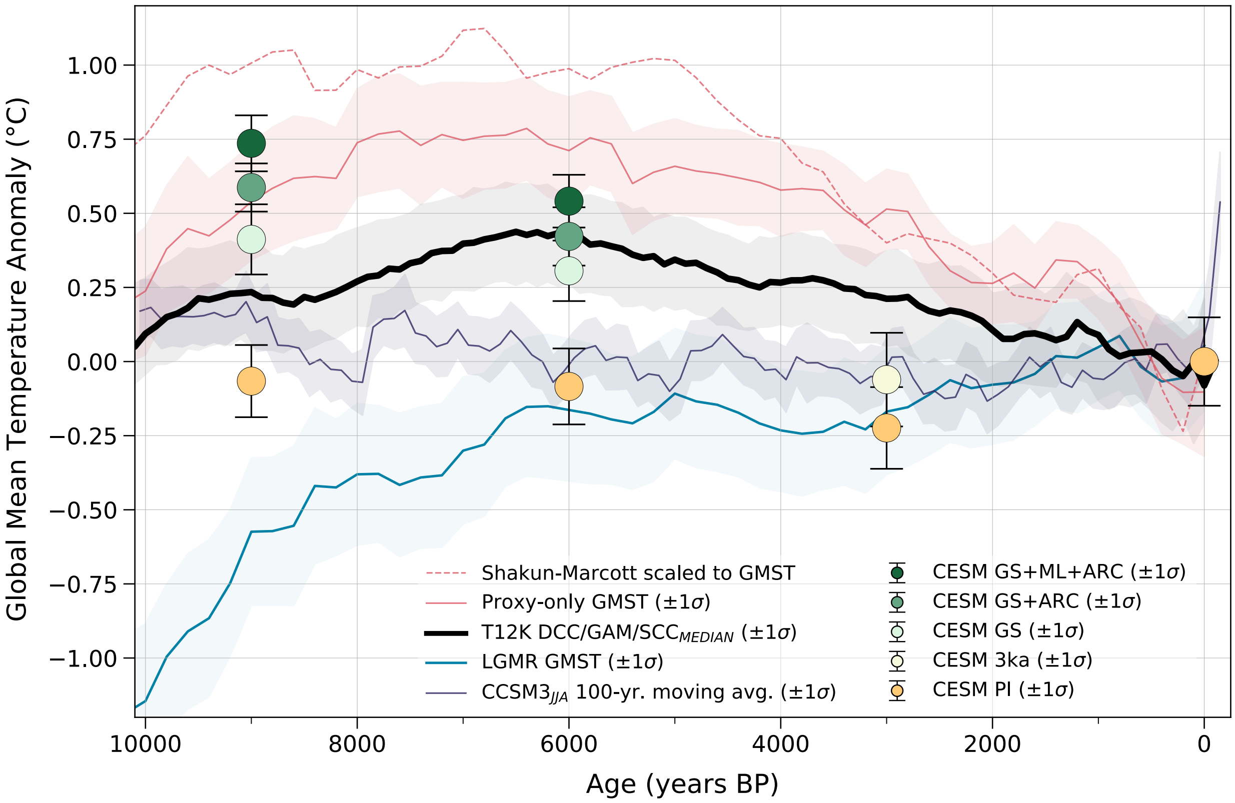

Figure S2. Model-proxy comparison of JJA global mean surface temperature anomalies. This plot is identical to Figure 1 in the main text, but displays boreal summer (JJA: June, July, August) average CESM1.2 and CCSM3 TraCE-21ka temperature anomalies and adds two proxy curves from (*12*): the Shakun-Marcott curve scaled to global mean surface temperature (dashed red line) and the proxy-only reconstruction of global mean surface temperature (red line ± one standard deviation).

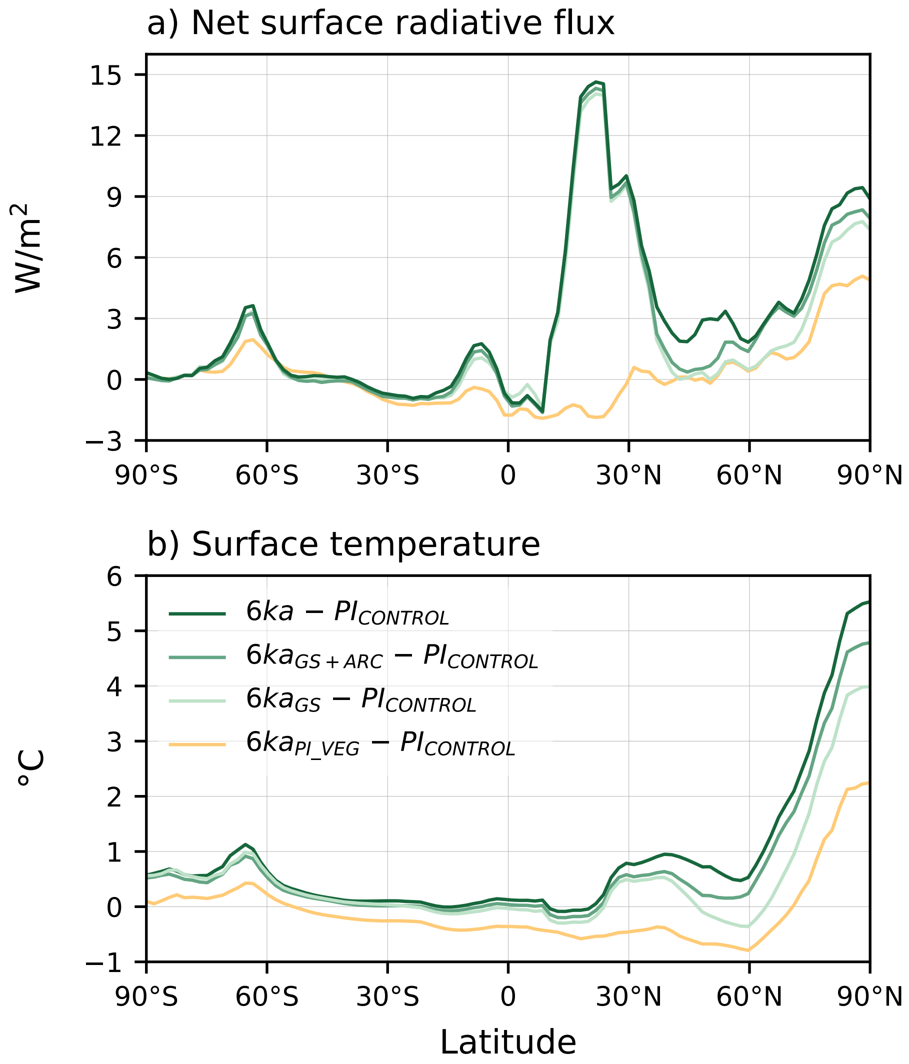

Figure S3. Contributions of radiative fluxes to surface warming. Annual zonal mean anomalies (relative to $PI_{CONTROL}$) of a) net surface radiative flux (shortwave–longwave) and b) surface temperature for 6 ka BP sensitivity experiments: $6ka_{PI\_VEG}$ (orange), $6ka_{GS}$ (light green), $6ka_{GS+ARC}$ (green), and *6ka* (darkest green).

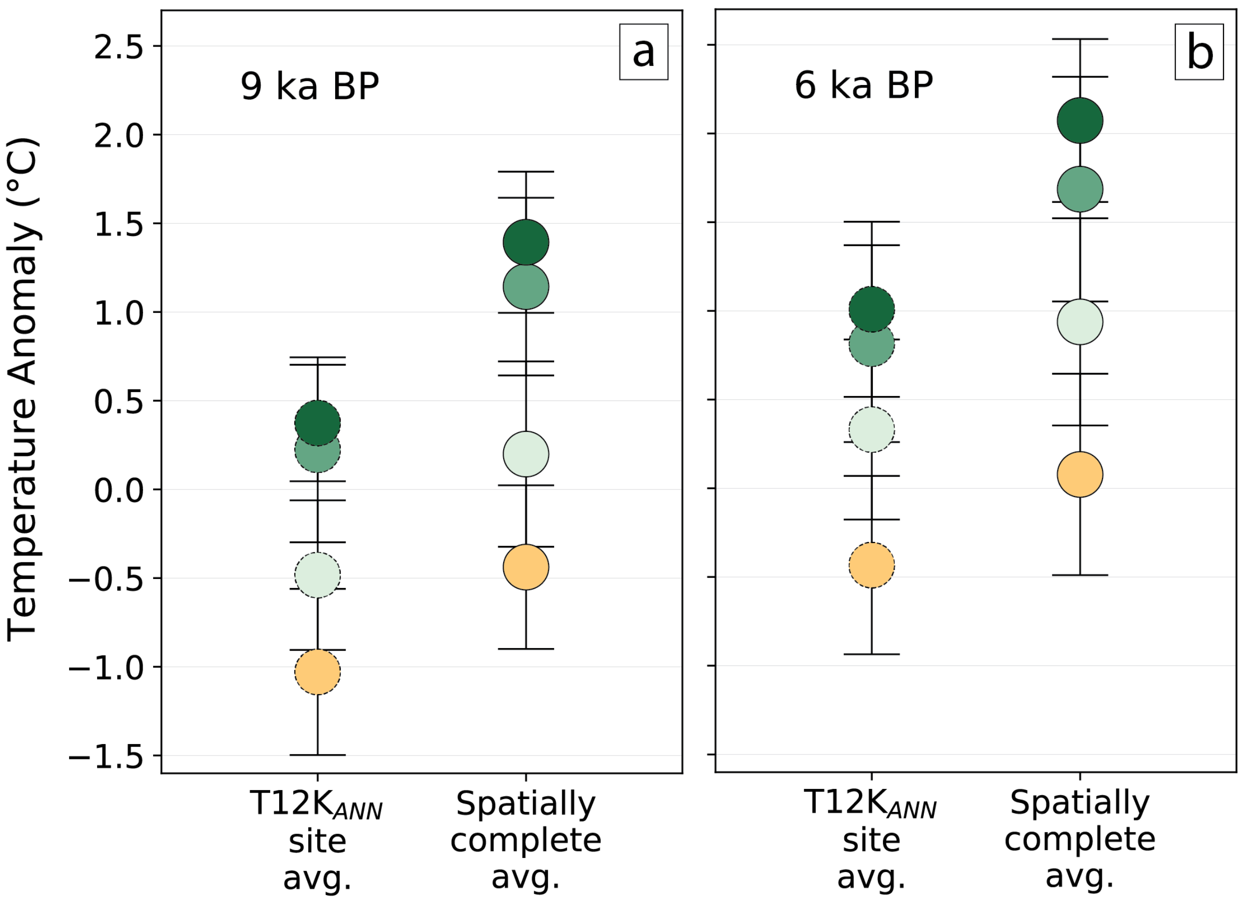

Figure S4. Comparison of simulated high-latitude temperature anomalies at 9 and 6 ka BP. Simulated CESM1.2 annual temperature anomalies averaged over 60–90°N for a) 9 and b) 6 ka BP. Shown on the left in each panel is the 60–90°N average (± one standard deviation) calculated only from model grid cells corresponding to the T12K$_{ANN}$ composite and on the right is the spatially complete region average (± one standard deviation). The spatially complete high latitude mean temperature anomalies are, on average, higher than the T12K$_{ANN}$-grid cell anomalies by ~0.8°C at both 9 and 6 ka BP.

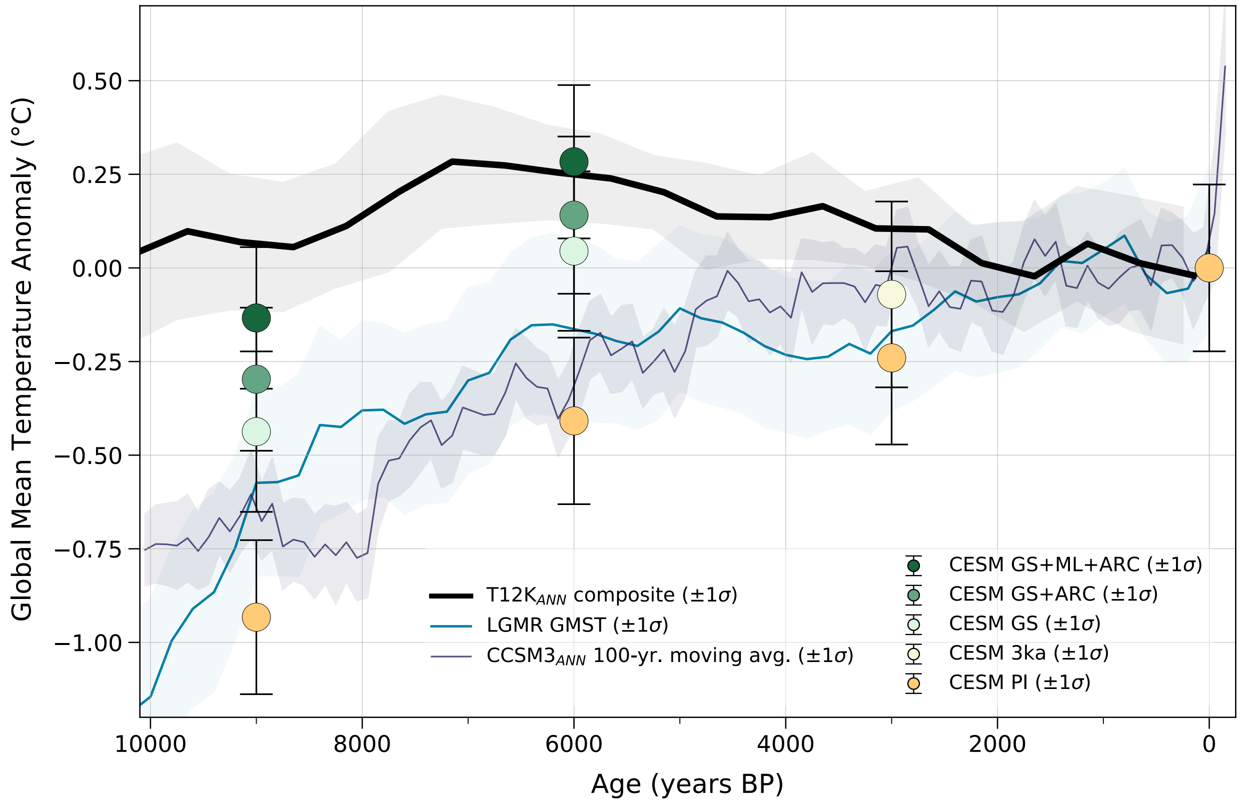

Figure S5. Model-proxy comparison of T12K$_{ANN}$ location mean surface temperature anomalies. This plot is identical to Figure 1 in the main text but shows the T12K$_{ANN}$ composite curve and calculates CESM1.2 temperature anomalies as the mean of only grid cells corresponding to the T12K$_{ANN}$ composite (*3*). Comparison between Figure 1 and this figure indicates that the global mean temperature anomalies in CESM1.2 (Figure 1) are higher than the anomalies from the average of sampled locations in the T12K$_{ANN}$ composite.

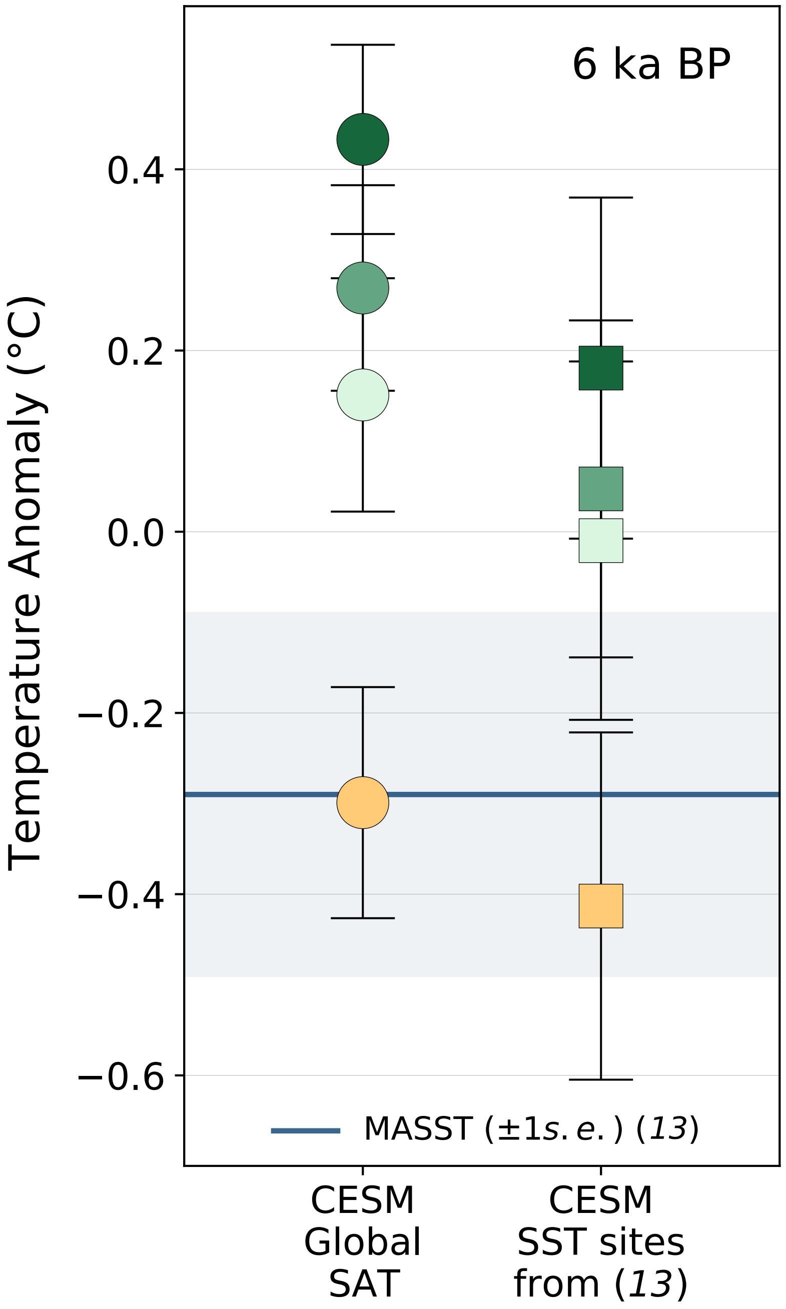

Figure S6. Comparison of simulated sea surface temperature at Bova et al. grid cells with global mean air temperature. Simulated CESM1.2 annual anomalies at 6 ka BP of global mean surface air temperature (SAT, circles; left) and mean sea surface temperature (SST, squares) of grid cells corresponding to Bova et al. (*13*) locations (right). The blue line signifies the 6 ka BP mean value ± one standard error from the SST reconstruction by Bova et al. (*13*). CESM1.2 colors are the same as Figure 1. The differences shown here between global mean SAT and Bova et al. (*13*) grid cell mean SST anomalies demonstrate that the Bova et al. (*13*) reconstruction is not representative of, and is colder than, global mean surface air temperature anomalies.

a) ***6ka*** and ***9ka***: In the African Sahara, shrub/C_4 grass replaces bare ground

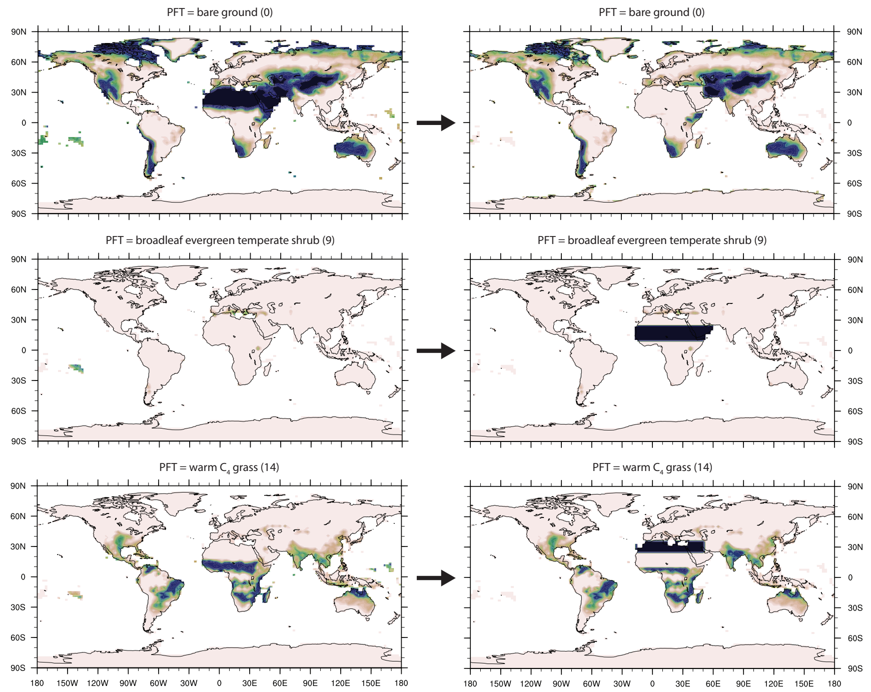

b) ***6ka*** and ***9ka***: In the Arctic north of 50°N, boreal forest replaces C_3 grass

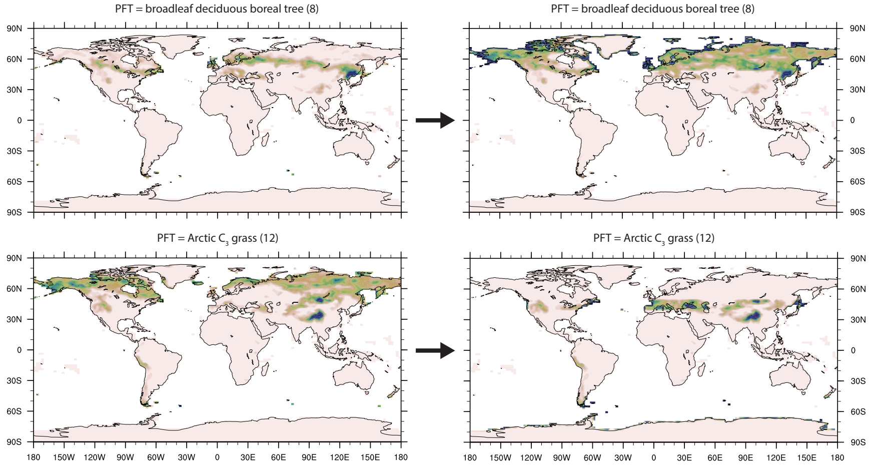

c) ***6ka*** and ***9ka***: In the mid-latitudes between 30°N and 60°N, deciduous forest replaces C_3 grass

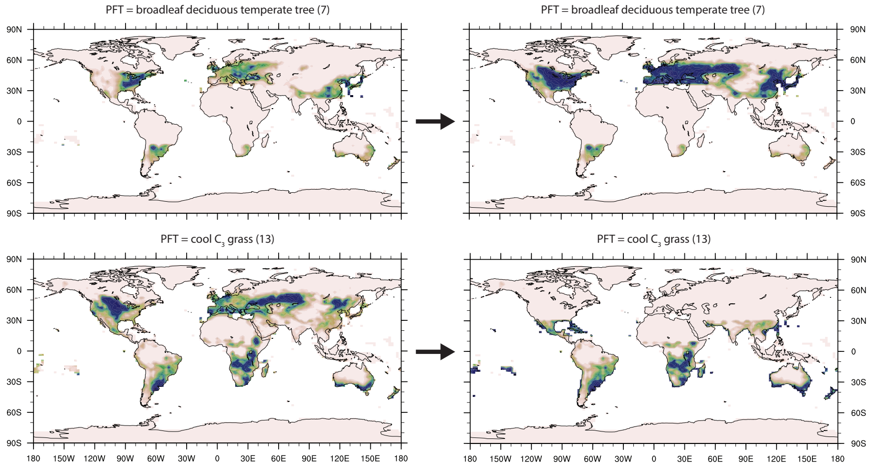

d) ***3ka***: Sahara/Sahel transition shifts ~5° north; between 40 and 60°N, deciduous forest replaces 50% of C_3 grass

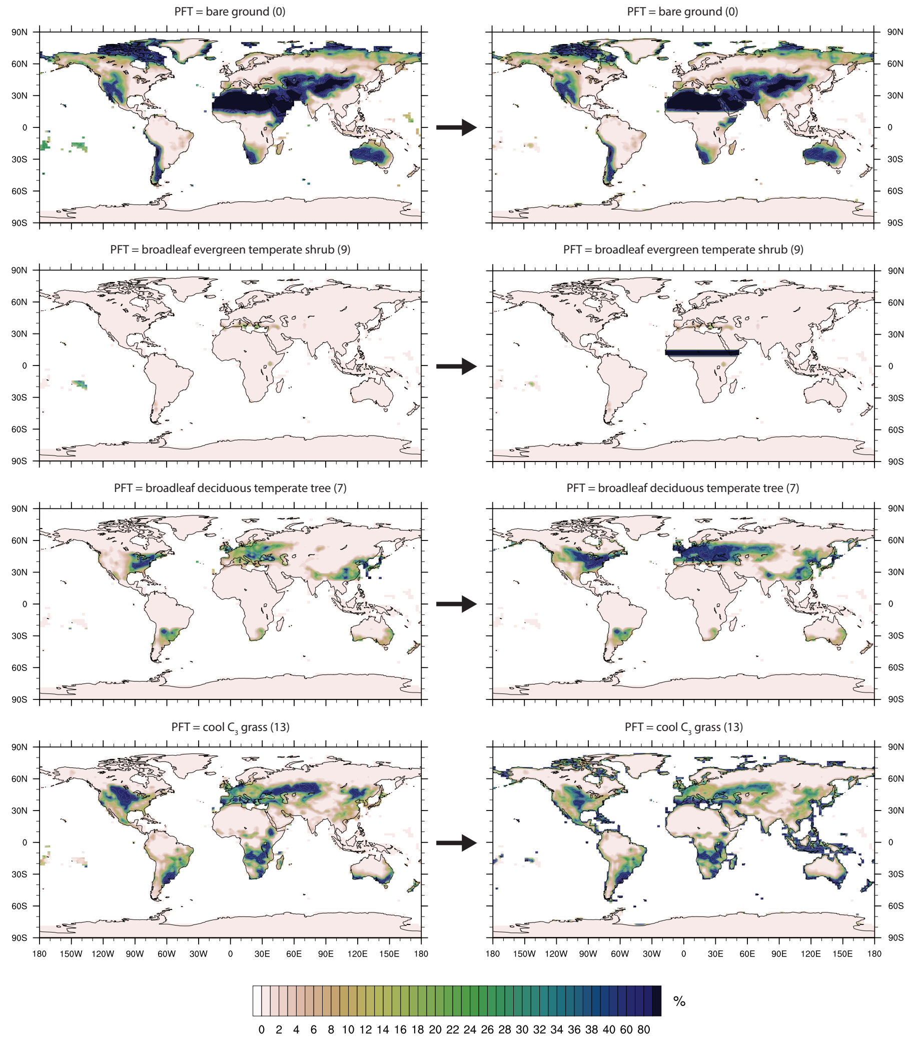

Figure S7. Modifications made to plant functional type. (a-c) *9ka* and *6ka* and (d) *3ka*. Numbers correspond to index for the specific plant functional type in CLM4.

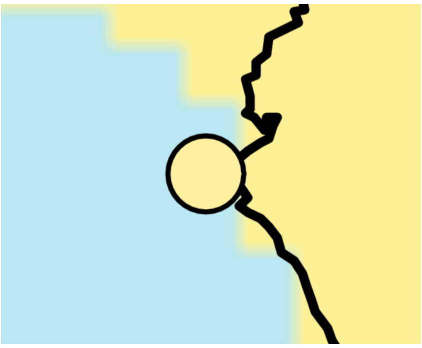

Figure S8. Example of adjacent cell agreement. This image depicts an example of agreement when calculating the percentage of nearest-neighbor grid cells where both the model and proxy record agree in sign of ΔT. In this example, the proxy record and the model grid cells directly adjacent to the east agree in sign of ΔT.

Table S1. List of all CESM1.2 simulations used in this study. Included are the corresponding colors used for each simulation, details regarding length and branch history, and specifications of orbit year, greenhouse gases, dust loading, and vegetation type in the Northern Hemisphere.

Simulation	*Years Run*	*Branched From*	*Orbit Year*	*CO_2 (ppm)*	*CH_4 (ppb)*	*N_2O (ppb)*	*Global dust (AOD*10^{-3})*	*Northern Hemisphere Vegetation*		
								Sahara	*Mid-latitudes*	*Arctic*
$PI_{CONTROL}$	900	N/A	1850 CE	284.7	791.6	275.7	23.3	Bare ground	C_3 grass	C_3 grass
$3ka_{PI\_VEG}$	900	N/A	3 ka BP	275.0	580.0	270.0	21.0	Bare ground	C_3 grass	C_3 grass
3ka	200	*$3ka_{PI\_VEG}$*	3 ka BP	275.0	580.0	270.0	20.4	Shrub to 16°N	50% deciduous forest	C_3 grass
$6ka_{PI\_VEG}$	400	*$6ka_{GS+ARC}$*	6 ka BP	264.4	597.0	262.0	20.1	Bare ground	C_3 grass	C_3 grass
$6ka_{PI\_VEG\_LOWDUST}$	200	*$6ka_{PI\_VEG}$*	6 ka BP	264.4	597.0	262.0	5.7	Bare ground	C_3 grass	C_3 grass
$6ka_{GS}$	200	*$6ka_{GS+ARC}$*	6 ka BP	264.4	597.0	262.0	4.1	Shrub/C_4 grass	C_3 grass	C_3 grass
$6ka_{GS+ARC}$	900	N/A	6 ka BP	264.4	597.0	262.0	4.1	Shrub/C_4 grass	C_3 grass	Boreal forest
6ka	200	*$6ka_{GS+ARC}$*	6 ka BP	264.4	597.0	262.0	4.9	Shrub/C_4 grass	Deciduous forest	Boreal forest
$6ka_{HIGHDUST}$	200	*6ka*	6 ka BP	264.4	597.0	262.0	99.9	Shrub/C_4 grass	Deciduous forest	Boreal forest
$9ka_{PI\_VEG}$	300	*$9ka_{GS}$*	9 ka BP	260.2	658.5	255.0	51.8	Bare ground	C_3 grass	C_3 grass
$9ka_{GS}$	900	N/A	9 ka BP	260.2	658.5	255.0	4.1	Shrub/C_4 grass	C_3 grass	C_3 grass
$9ka_{GS+ARC}$	250	*$9ka_{GS}$*	9 ka BP	260.2	658.5	255.0	4.2	Shrub/C_4 grass	C_3 grass	Boreal forest
9ka	250	*$9ka_{GS+ARC}$*	9 ka BP	260.2	658.5	255.0	4.9	Shrub/C_4 grass	Deciduous forest	Boreal forest

Table S2. Top-of-atmosphere shortwave radiative responses to vegetation change. Changes are W/m^2 relative to $6ka_{PI\_VEG}$ calculated with the approximate partial radiative perturbation (APRP) method (*42*). α: surface albedo radiative response to vegetation change (Eq. 16a); *cld:* cloud radiative response to vegetation change (Eq. 16b); and *clr*: non-cloud atmospheric constituent radiative response to vegetation change (Eq. 16c).

Experiment	α	*cld*	*clr*
6ka	+2.41	–0.61	–0.02
$6ka_{GS+ARC}$	+2.11	–0.71	–0.04
$6ka_{GS}$	+1.91	–0.79	–0.05

Data File S1 (Microsoft Excel format). Full 9 and 6 ka BP statistical analysis results for quantifying improvements in model-data agreement.
